# Fabrication of ordered nanoporous anodic alumina prepatterned by mold-assisted chemical etching

**DOI:** 10.1186/1556-276X-6-157

**Published:** 2011-02-21

**Authors:** Kuan-Liang Lai, Min-Hsiung Hon, Ing-Chi Leu

**Affiliations:** 1 Department of Materials Science and Engineering, National Cheng Kung University, Tainan 701, Taiwan; 2 Department of Materials Science, National University of Tainan, Tainan 700, Taiwan

## Abstract

In this article, a simple and cost-effective method to create patterned nanoindentations on Al surface via mold-assisted chemical etching process is demonstrated. This report shows the reaction-diffusion method which formed nanoscale shallow etch pits by the absorption/liberation behaviors of chemical etchant in poly(dimethylsiloxane) stamp. During subsequent anodization, it was possible to obtain the ordered nanopore arrays with 277 nm pitch that were guided by the prepatterned etch pits. The prepatterned etch pits obtained can guide the growth of AAO nanopores during anodization and facilitate the preparation of ordered nanopore arrays.

## Introduction

In recent years, nanoporous anodic aluminum oxide (AAO) has become a popular template system for the synthesis of various functional nanostructures which have extensive applications in scientific and commercial fields [1-4]. In the syntheis of template-based materials, the template with long-range-ordered nanostructure is attractive, in order that structurally well-defined materials can be consequently produced. In general, Al anodization processes, highly regular arrangement of pores, however, occurs only within a small process window, and the domain size (ordering length) is usually limited to a micrometer scale on Al foils [5,6]. In order to achieve an ordered pore arrangement over a larger area, Masuda et al. [5,7] developed a pretexturing process of Al using nanoimprinting with a SiC mold. Shallow indentations on an Al substrate initiate pore nucleation during anodization and lead to a long-range-ordered pore arrangement within the stamped area.

Self-ordered and prepatterned guided growths are two kinds of anodization technology, which are competing in the aspects of product quality and production cost. For prepatterned guided anodization, imprinting methods have been used by several author groups to prepare ordered AAO, wherein nanoindentations are created by transferring patterns from hard master stamp onto the Al surface under a high pressure (5-25 kN cm^-2^) before anodization [8-10]. Despite the ideally ordered patterns obtained, this method is limited by the pattern transfer protocol, and pattern transferred by imprint lithography directly onto metallic substrates such as Al foils or Al films requires 50-2000 times higher pressures in comparison with imprint lithography on polymer layers [11]. The applied pressure for pattern transfer tends to crack the substrates underneath the Al films, such as silicon and glass with brittle property, and leads to substrate fracture. Otherwise, damage to the imprint stamp often occurs after several runs of imprinting because of the high mechanical stresses.

In the reported literatures, some outstanding methods, such as focused ion beams [6], optical diffraction gratings [12], colloidal lithography [13], block-copolymer self-assembly [14], and metal mask [15] were also used to achieve prepatterning of Al substrates, thus avoiding fabrication of the expensive hard imprint stamp. However most of them have limitations in scalability or size of ordered domains. Consequently, a simple and economic method for realization of a long-range-ordered AAO over very large areas (cm^2 ^to wafer size) still faces challenges. Recently, some methods, such as guided electric field method [16], and step and flash imprint lithography [17], have been developed to fabricate wafer-scale-ordered AAO.

Ideally, a simple and cost-effective process for preparing ordered AAO should combine with a high-throughput method to create patterned nanoindentations on Al surface. It should also be substrate-friendly to avoid damaging the substrate such as thin Al film-deposited Si.

The reaction-diffusion wet stamping (RD-WETS) method uses a nanopatterned agarose stamp such as poly(dimethylsiloxane) (PDMS) in soft lithography. An agarose stamp soaked with an appropriate chemical reactant can etch/dissolve the desired hard material by simply contacting with the substrate (e.g., HF for SiO_2 _or HCl/FeCl_3 _for Cu) [18-20]. Localized etching is mediated by a mold-assisted chemical etching initiated from the stamp microfeatures, and excellent uniformity over areas of several square centimeters can be achieved.

In this study, a simple and reliable method for substrate prepatterning by soft imprinting, using a diffusion-reaction-controlled wet chemical etching method, is developed thus avoiding the use of sophisticated device fabrication procedures. In addition, the highly ordered porous alumina on Al foils with the help of prepatterned indentations by the above-mentioned wet stamping were fabricated.

## Experimental section

The master molds for PDMS stamp fabrication were sub-micromter gratings (for 1D pattern) and Si wafers with regular pit arrays (for 2D pattern). The membrane stamp was made by pouring a mixture of PDMS prepolymer (Dow Corning Sylgard 184) and its curing agent (10:1 by weight) into the masters, which was cured for 1 h at room temperature and then for 4 h at 60°C in an oven. The PDMS stamps about 2 mm in thickness were replicated from straight line diffraction grating surface (Thorlabs, Inc. 3600 and 1800 lines/mm), and Si mold with regular pit arrays of 277-nm pitch. The flexible agarose membrane has a better attachment to solid surface. Al samples with a total surface area of 2 × 2 cm^2 ^were cut from an aluminum sheet (99.99%, Alfa Aesar), degreased in acetone and dried.

The Al sheet was electropolished at a constant voltage in perchloric acid/ethanol (1:4 V/V ratio) at 4°C for 30 s, to diminish the roughness of Al foil surface. Patterns on Al substrate were etched using a mold previously soaked in a diluted solution of mixed acid (2%) in alcohol (mixed acid composition: 0.15 M HNO_3_, 0.6 M H_3_PO_4_, and 0.2 M CH_3_COOH). The nitric acid consumes some of the aluminum material to form an aluminum oxide layer. This oxide layer is then dissolved by the phosphoric acid, and more Al_2_O_3 _is formed to keep the oxidation/dissolution cycle going. The diluted etchants moderated the condition of etching reaction and contributed to the formation of nanopatterns. The PDMS stamp was soaked in etching solution for 10 min and absorbed in the latter, and the time period for etching process was within 5 min. After nanoindentation by the RD-WETS process with PDMS membrane stamps, anodization was conducted under a constant voltage in phosphoric acid solution. The ordered AAO structures were examined by scanning electron microscopy (SEM, Hitachi S3000) and atomic-force microscopy (AFM, Digital Instrument Nanoscope LFM-3).

## Results and discussions

**Figure 1:**
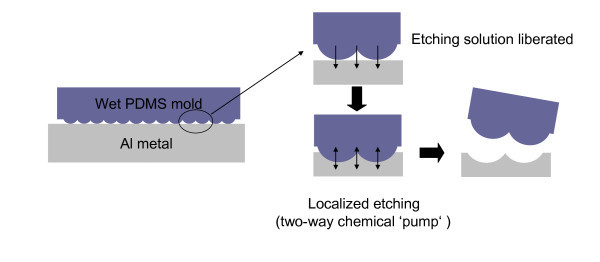
**Scheme of the experimental procedures for reaction-diffusion wet etching**.

The RD-WETS approach can be extended to structuring hard materials by chemical etching reaction. Regardless of the substrate type, the mechanism of localized microetching relied on the diffusive transport of chemicals within a stamp [18-20]. Figure 1 shows the scheme of mold-assisted microetching of substrate. The PDMS stamp was soaked in etching solution (2% mixed acid in alcohol) for 10 min and absorbed approximately 4% etching solution, and the residual solution on the surface of stamp was removed by N_2 _flow. Then, the wet stamp was set on Al substrate with a slight loading (0.01 MPa) to ensure a conformal contact with substrate. The etchant-contained alcohol liberated from stamp reacted with Al metal, and the reaction products diffused into PDMS along the concentration gradient as the arrows indicated. Compared with the conventional RD-WETS process, this method used alcohol in place of water because the alcohol in agarose mold has a higher absorptivity than water [21]. It helps to adjust the degree of reaction-diffusion by the solvent liberation/absorption process and this two-way chemical "pump" increases the work efficiency. From this point of view, the parameters of RD process should be adjusted to meet the requirements of imprinting nanopatterns on Al surface. In general, the shallow nanoscale concave (just 3 nm in depth is sufficient) can guide the ordered growth of AAO effectively [9].

**Figure 2:**
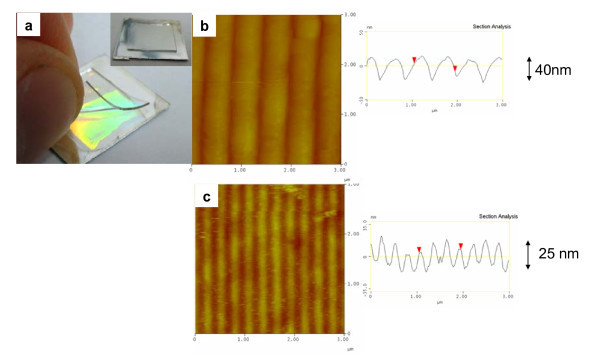
**The photograph and AFM images of the aluminum substrate with grating prepatterns (a) sample after RD-WETS**. **(b) **procedure with pitch of 555 nm; **(c) **277 nm.

The photograph of sample after RD-WETS is shown in Figure 2a, where the Al surface with grating prepattern appears under visible diffractive light and results in a uniform prepattern over large areas (up to 2 × 2 cm^2^). A detailed investigation of the film topography was performed by AFM as Figure 2b,c shows. The pitches of grating patterns are 555 and 277 nm with pattern heights of 40 and 25 nm, respectively. Overall, the reaction-diffusion process allowed the PDMS to cut into the Al substrate, in particular, with retention of the stamp's topography.

**Figure 3:**
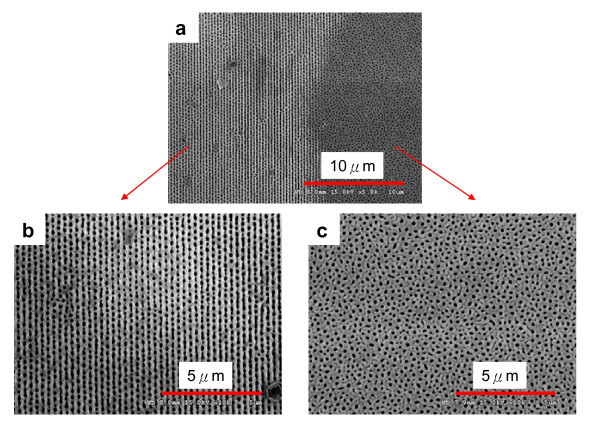
**SEM micrographs of anodization sample (a) alumina pores obtained from aluminum foils**. **(b) **alumina pores grown in the 1D grating-patterned area. **(c) **alumina pores grown in the unpatterned area. Anodization conducted in 0.3 M H_3_PO_4 _at 110 V and 5°C.

After the RD process, anodization was conducted under a constant voltage of 110 V in 0.3 M H_3_PO_4 _at 5°C. The anodization voltage for the prepatterned aluminum substrate was chosen to satisfy the linear relationship between the interpore distance and the anodization potential (2.5 nm/V^-1^) reported for the common anodization process [22]. Figure 3 shows SEM micrographs of alumina pores obtained from aluminum foils, half of which (left-hand side) were obtained on Al pretextured by RD-WETS. Pores arranged in a 1D grating configuration were observed only in the pretextured area, while the disordered pores were found in the untreated area. In addition, it was found that the PDMS stamp can well tolerate the dilute acid etchant, which implies that the soft stamp can be reused multiple times without noticeable decrease in patterning quality [18].

**Figure 4:**
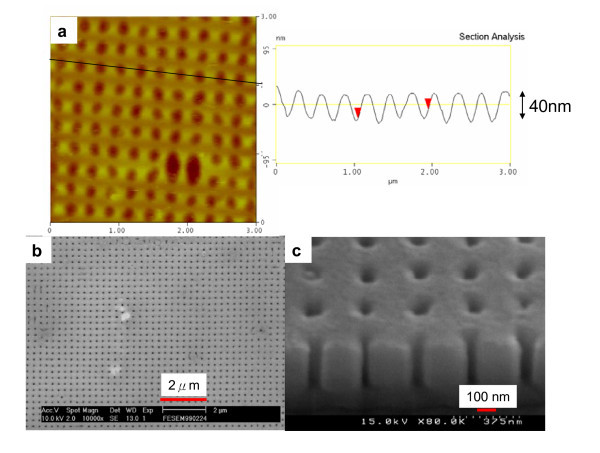
**AFM and SEM images of Al prepattern and AAO (a) 2D Al prepatten after RD-WETS**. **(b, c) **2D prepattern-induced regular AAO array. Anodization conducted in 0.3 M H_3_PO_4 _at 110 V and 5°C.

Furthermore, the 2D periodic prepattern on Al was fabricated using a PDMS mold with square dot arrays, as Figure 4a shows. Shallow etched pits in the prepattern (approximately 40-nm depth) serves as nucleation sites for the development of a pore in the early stage of anodization [5-7], and results in the eventual growth of a pore channel. The results shown in Figure 4b confirm that the predetermined pattern can act as initiation points and guide the growth of channels in the oxide film. Straight oxide nanochannels (Figure 4c) with uniform-sized pores are obtained.

**Figure 5:**
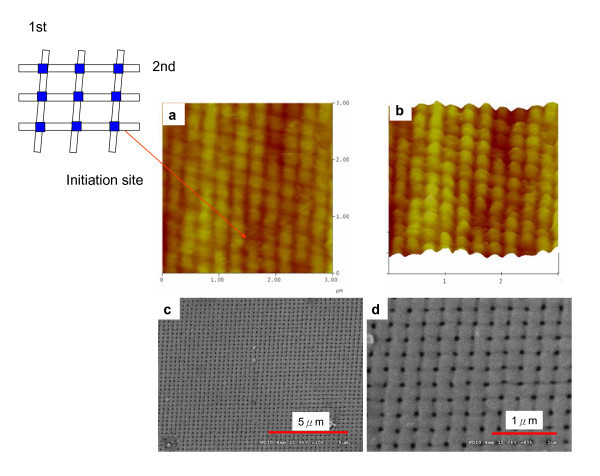
**AFM and SEM images of Al prepattern and AAO (a, b) Al prepattern featuring a second grating on a primary structure with ~85° rotation and pitch of 277 nm**. **(c, d) **prepattern-induced regular AAO array. Anodization conducted in 0.3 M H_3_PO_4 _at 110 V and 5°C.

Furthermore, the two-step imprinting was used to fabricate multiple patterns from a single master. The two-step imprinting can be used to selectively etch Al at established primary structure because the etchant only acts at the contact site between the mold and substrate [18]. After the first mold-assisted etching, a second etching step was performed using the same grating rotated by approximately 85° around the axis perpendicular to the surface to discriminate this multiple case from one-step imprinting method. A parallelogram profile of etched pit arrays was obtained, as illustrated in Figure 5a,b. From the AFM images, the intersects of grating pattern show shallow indent arrays which resemble point-like depressions [5,12] and have just several nanometers in depth relative to the local surface around them. In addition, the double-etching sites serve as the nucleation sites, and the ordered AAO growth can be maintained as shown in Figure 5c,d. A single pore just appears on double-etching site and the notches of multiple etching remain on the AAO surface and parallelogram (i.e., non-right angle) patterns of pore arrays are obviously different from the directly imprinted 2D square prepatterns (Figure 4b). All of these experimental findings suggest that this mold-assisted etching method is industrially applicable to a large-scale production of nanopatterning and has the potential of achieving the aim of fabricating nanostructured functional AAO with required design geometry.

## Conclusions

In conclusion, a novel method for fabricating prepatterned Al foil was developed, which used the reaction-diffusion process mediated by a PDMS template. By means of using the diluted (2%) mixed acid solution as a chemical etchant, the wet soft stamp can indent nanoscale shallow concaves on aluminum without the need of excessive loading. Furthermore, based on the phenomenon of multiple RD-WETS imprinting, 2D prepattern by multiple etching could be made using simple stripe-patterned stamps with selected orientation. After anodization, a uniform, ordered AAO array with 277-nm interpore distance guided by the prepattern was obtained. Combining mold-assisted chemical etching and anodization reaction, this process provides a simple and efficient route to obtain ordered nanostructures for further nanodevice applications.

## Abbreviations

AAO: anodic aluminum oxide; PDMS: poly(dimethylsiloxane); RD-WETS: reaction-diffusion wet stamping.

## Competing interests

The authors declare that they have no competing interests.

## Authors' contributions

MHH and ICL planned and supervised the research project. ICL, KLL and MHH conceived and designed the experiments. KLL carried out the experiments, analyzed the data, and drafted the manuscript. ICL participated in the analysis of experimental data and the writing of manuscript. All authors discussed the results and commented on the manuscript.
